# No.I. The Psychological Vocation of the Physician

**Published:** 1854-01-01

**Authors:** 


### THE PSYCHOLOGICAL VOCATION OF THE PHYSICIAN.

HAVING had the honour of being appointed Lettsomian Professor of Medicine, of the *Medical Society of London*, I accepted the office with, I trust, a humble appreciation of my capacity to discharge satisfactorily its duties in a manner commensurate with their grave importance. The distinction conferred upon me is one not to be lightly esteemed. To be selected from a body of physicians of great and admitted eminence, of profound learning, of scientific attainments of a high order, of undoubted eloquence, as Lettsomian lecturer on medicine, is an event which I shall ever cherish with lively emotions, as one of the most pleasing and personally gratifying occurrences in my chequered life.

The post thus assigned to me by your kindness, entails upon me the pleasing duty of delivering, before this Society, *three* lectures connected with that division of medical science with which the physician is supposed to be especially conversant. In selecting three topics for illustration, I felt anxious to bring under review subjects worthy of your consideration; and involving in their elucidation, points of theoretical as well as of practical interest. I flattered myself that I should be realizing your anticipations, and be acting in unison with the wishes of the Council, if I were to confine myself to the exposition of three points connected with those investigations with which my own mind is supposed to be more particularly occupied. Under this impression, I have, with, I hope, a right sense of the difficulty of my self-imposed task, selected, as the subject of my first and introductory lecture, a question of extreme interest to all engaged in the practice of medicine.

Having, from an early period of my professional studies, formed a high estimate of the importance of the science of mental philosophy, and devoted much attention to the investigation of questions relating to the influence of the spiritual upon the material portions of the organization, I may, perhaps, be excused, if I should, in the course of my remarks upon the necessity of a more general and accurate knowledge of the science of mind, convey a somewhat extravagant conception of the value of that section of inquiry which presents so many charms to my own imagination. With the object of demonstrating the theoretical and practical advantages resulting from this investigation, I have undertaken, in my first lecture, to illustrate the *special psychological attributes of the physician*—to claim for the cultivators of medical science higher and more exalted functions than those usually assigned to them—to consider the physician in his *spiritual* character, as having at his command, and under his control, a *medicina mentis* as well as a *medicina corporis*—agents of great power and magnitude—which have not been sufficiently recognised or appreciated. It will be my object to establish the close connexion between the SCIENCE OF MIND, and the SCIENCE AND PRACTICE OF MEDICINE, and to illustrate the true philosophic character of the professors of the healing art. "Δει μεταγειν την σοφιαν εἰς την ἰατρικην, και την ἰατρικην εἰς την σοφιαν· ἰατρος γαρ φιλοσοφος ἰσοθεος."*

We form but a low and grovelling estimate of our high destination—of the duties of our dignified vocation, *if we conceive that our operations are limited to a successful application of mere* PHYSICAL AGENTS. God forbid that we should thus vilify ourselves, and degrade our noble science! "A physician whose horizon is bounded by an historical knowledge of the human machine, and who can only distinguish terminologically and locally the coarser wheels of this piece of intellectual clockwork, may be, perhaps, idolized by the mob; but he will never raise the Hippocratic art above the narrow sphere of a mere bread-earning craft."† The physician is daily called upon, in the exercise of his profession, to witness the powerful effect of mental emotion upon the material fabric. He recognises the fact, although he may be unable to explain its rationale. He perceives that moral causes induce disease, destroy life, retard recovery, and often interfere with the successful operation of the most potent remedial means exhibited for the alleviation and cure of bodily disease and suffering. Although such influences are admitted to play an important part, either for good or for evil, I do not conceive that, as

* Hippocrates. † Schiller.

physicians, we have sufficient appreciation of their great importance.

"If a patient dies," says M. Reveillé-Parise, "we open his body, rummage among the viscera, and scrutinize most narrowly all the organs and tissues, in the hope of discovering lesions of some one sort or another; there is not a small vessel, membrane, cavity, or follicle, which is not attentively examined; the colour, the weight, the thickness, the volume, the alteration—nothing escapes the eye of the studious anatomist. He handles, touches, smells, and looks at everything; then he draws his conclusions one way or another. One thing only escapes his attention; that is, he is looking at merely organic effects, forgetting all the while that he must mount higher up to discover their causes. These organic alterations are observed, perhaps, in the body of a person who has suffered deeply from mental distress and anxiety; these have been the energetic cause of his decay, but they cannot be discovered in the laboratory or the amphitheatre. Many physicians of extensive experience are destitute of the ability of searching out and understanding the moral causes of disease; they cannot read the book of the heart, and yet it is in this book that are inscribed, day by day, and hour by hour, all the griefs, and all the miseries, and all the vanities, and all the fears, and all the joys, and all the hopes of man, and in which will be found the most active and incessant principle of that frightful series of organic changes which constitute pathology. This is quite true—whenever the equilibrium of our moral nature is long or very seriously disturbed, we may rest assured that our animal functions will suffer. Many a disease is the *contre-coup*, so to speak, of a strong moral emotion; the mischief may not be apparent at the time, but its germ will be nevertheless inevitably laid." *

In proportion as we recognise our psychological character and position, and estimate the effect of these spiritual agents, shall we be successful at the bedside, elevate ourselves in the social scale, and not only deserve, but command, the respect of the public, and place the science of medicine upon the highest vantage-ground of which it is susceptible. How is it possible for us to influence the minds of others if we have no accurate knowledge of the constitution and operation of our own understandings? As well might the physician administer, for the

* "On Moral Therapeutics." Paris.

relief of an acute malady, a material agent of whose properties and *modus operandi* he is avowedly ignorant.

> "He that would govern others, first should be
> The master of himself, richly endued
> With depth of understanding, height of knowledge."
> MASSINGER.

Referring generally to the present aspect of that branch of philosophy whose claims I am now advocating, I would, *in limine*, observe, that the advancement of mental science has of late years been greatly retarded by the prejudices which have prevailed in reference to all abstract metaphysical investigations. An impression has existed, that this inquiry unfitted the mind for the contemplation of subjects more immediately associated with the useful and practical affairs of every-day life; that the researches of metaphysicians served only to darken, bewilder, and dazzle the understanding, and to teach the use of pedantic jargon, and of obscure and transcendental phraseology. Hence arose the sarcasm, that to recommend a person to engage in the study of metaphysics was a delicate and indirect mode of suggesting the propriety of subjecting him to the restraint of an asylum. "I am the person you wish to see," said the illustrious Plato to his foreign guests, who desired an introduction to the grave philosopher, under the impression that they were to see a man exhibiting qualities very different from those possessed by ordinary mortals. Does, I would ask, the mind grow severe in proportion to its enlightenment? Why should a knowledge of the most exalted department of philosophy unfit us for the active pursuits of life, or for the society of mankind? Need we be surprised at the attempts which have been made, in the present utilitarian age, to depreciate the study of metaphysical philosophy, when we take a retrospective glance at its history. The modern metaphysician is engaged in more useful and loftier speculations than that of considering whether the *essence* of mind be distinct from its *existence*, and what are the qualities inherent in it as a nonentity? Whether angels passed from one point of space to another without passing through the intermediate points? Whether they can visually discern objects in the dark? Whether more than one angel can exist at the same moment in the same physical point? Whether they can exist in a perfect vacuum, with any relation to the absolute incorporeal void? Whether, if an angel were *in vacuo*, the void could still be termed perfect? These, and similarly abstruse and absurd speculations, seriously occupied the patient attention of a few of the learned schoolmen and theologians of former times, and gave rise to the idea of the science of metaphysics being the art of talking grave nonsense upon subjects beyond the limits of the human understanding. We are not justified, however, in any wholesale condemnation of these apparently profitless and Quixotic speculations. May we not use the language of the founder of the Inductive Philosophy, and say of the ancient schoolmen, that "in seeking for brilliant impossibilities they sometimes discovered useful realities."

Bacon, when referring to the researches of the alchemists for the philosopher's stone, says, that they performed the office of the husbandman, who, in seeking for a hidden treasure, turned up the soil, and pulverized the earth, thereby rendering it better fitted for the purposes of vegetation. Although the schoolmen were baffled in their attempts to discover the essence of the soul, and to ascertain with any degree of satisfaction to their own minds the precise number of angelic spirits who could *pirouette* at the same instant upon the point of a needle, they nevertheless opened a path for the philosopher, amidst the dreary forest which he had to traverse, and pointed out to him the dangerous portions of his journey, in which they themselves had stumbled and fallen.

Modern Metaphysics, and its sister science, Theology, hold the same relations to the rhapsodies of the schoolmen as modern chemistry does to the speculations of the alchemist. No right-thinking men would repudiate the study of modern chemistry on account of the obscure and apparently profitless researches of the alchemists: by parity of reasoning, are we justified in denouncing the serious and patient study of mental philosophy, on account of the scholastic jargon, nonentities, unmeaning generalities, and inanities, of some of the ancient metaphysicians?

In forming an estimate of the value of any branch of philosophical inquiry, we must be cautious how we apply the interrogatory, *cui bono?*—neither must we adopt as our model of imitation the mathematician, who, refusing to admit that any advantage could result from the study of a science not directly related to his own favourite study, exclaimed, when recommended to read Milton's "Paradise Lost," "What does it prove?"

Are the lofty emotions, the glorious imagery, the sublime speculations, the melodies that have charmed our ear, elevated our thoughts, improved our hearts, ennobled our nature, purified our manners, and thrown rays of sunshine over the dreary and thorny path of life, to be dismissed from our contemplation because they have no obvious and direct relationship to the practical business of life? Let us not encourage the vulgar prejudice against those exalted inquiries that have no apparent or intimate association with the science of medicine, which constitute the charm and poetry of life, and exercise a powerful influence upon the intellectual progress of nations, the civilization of the world, and the character, happiness, and destiny of man!

"——— Desolater! who shall say
Of what thy rashness may have reft mankind?
Take the sweet poetry of life away,
And what remains behind?"

Goëthe, when referring to the healthful influences of imaginative literature upon the heart and intellect, eloquently observes,—"When the man of the world is devoting his days to wasting melancholy for some deep disappointment, or in the ebullience of joy is going out to meet his happy destiny, the lightly-moved and all-conceiving spirit of the poet steps forth to be the sun from night to day, and, with soft transitions, tunes his harp to joy or woe. From his heart, its native soil, springs up the lovely flower of wisdom; and if others, while waking, dream, and are pained with fantastic delusions from their every sense, he passes the dream of life like one awake, and the strangest incidents are to him a part both of the past and of the future. And thus the poet is at once a teacher, a prophet, and a friend of gods and men. At the courts of kings, at the tables of the great, beneath the windows of the fair, the sound of the poet was heard, when the ear and soul were shut to all beside; and men felt as we do when delight comes over us, and we pause with rapture, if, among dingles we are crossing, the voice of the nightingale starts out touching and strong. The poets found a home in every habitation of the world, and the lowliness of their position exalted them the more. The hero listened to their songs, and the conqueror of the earth did reverence to the poet, for he felt that without poets his own wild and vast existence would pass away and be forgotten for ever."*

* Wilhelm Meister.

Would that I could, in equally lofty, fervid, and touching eloquence, impress upon others the conception which I myself have formed of the value—the practical importance—to the physician, of a more general acquaintance with those branches of polite literature which serve to chasten the taste, discipline the mind, develop holy aspirations after truth, invigorate the understanding, improve the heart, and keep in abeyance those corroding emotions which often embitter our existence, engender disease, and shorten the duration of human life. The science of mind has been truly designated "*the science of ourselves*," of all that surrounds us, "of everything which we enjoy and suffer, or hope and fear; so truly the science of our very being, that it would be impossible for us to look back on the feelings of a single hour without constantly retracing phenomena that have been there, to a certain extent, the subject of our analysis and arrangement. The thoughts and faculties of our intellectual frame, and all which we admire as wonderful in the genius of others; the moral obligation which, as obeyed or violated, is ever felt by us with delight or with remorse; the virtues of which we think as often as we think of those whom we love, and the vices we ever view with abhorrence or with pity; the traces of divine goodness, which never can be absent from our view, because there is no object in nature which does not exhibit them; the feeling of our dependence upon the gracious Power that formed us; and the anticipation of the state of existence more lasting than that which is measured by the few beatings of our feeble pulse,—these, in their perpetual recurrence, impress upon us the vast importance of a knowledge of the philosophy of the human mind."*

When referring to the influence of such studies upon the mind, Burke, with great eloquence and truth, observes, that "whatever progress may be made towards the discovery of truth in this matter, we shall not repent the pains we have taken in it. The use of such inquiries may be very considerable. Whatever turns the soul inward on itself, tends to concentre its forces and to fit it for greater and stronger flights of science. By looking into physical causes, our minds are opened and enlarged, and in this pursuit, whether we take, or whether we lose our game, the chase is certainly of service. Cicero, true as he was to the academic philosophy, and consequently led to reject the certainty of phy-

* Browne.

sical as of every other kind of knowledge, yet truly confesses its great importance to the human understanding : '*Est animorum ingeniorumque nostrorum naturale quoddam quasi pabulum consideratio contemplatioque naturæ.*' If we can direct the light we derive from such exalted speculations upon the humbler field of the imagination, whilst we investigate the springs and trace the courses of our passions, we may not only communicate to the taste a sort of philosophical dignity, but we may reflect back on the severer sciences some of the graces and elegancies of taste, without which, the greatest proficiency in those sciences will always have the appearance of something illiberal."

This science, apart altogether from its direct utility, has other great and obvious advantages, which, in the absence of more conclusive recommendations in its favour, ought to demonstrate to us the importance and value of a knowledge of our own mental constitution. The discipline—the training—the expansion—which the mind undergoes in the study of its own operations, are of themselves benefits not lightly to be appreciated. The cultivation of habits of accurate observation and reflection, of patient attention, of rigid induction, of logical ratiocination, qualifies the mind for the more ready pursuit of those branches of knowledge that are considered to be more closely connected with the practical and active business of life. The mental *gymnasium* to which I refer is admirably fitted for the development, regulation, and cultivation of those faculties of the mind upon the right exercise of which depends our intellectual advancement and happiness.

It is not my wish, in advocating the claims of mental philosophy, to undervalue those sections of knowledge which have an almost exclusive reference to the *physical* sciences. I am quite disposed, however, to admit that it is an unfortunate effect of mere physical inquiry, when exclusively directed to the properties of external things, to render the mind in our imagination subordinate to the objects to which it is directed; the faculties are nothing, the objects are everything. The very nature of such inquiry leads us perpetually without to observe and arrange, and nothing brings us back to the observer and arranger within; or if we do occasionally cast an inquisitive glance on the phenomena of our thought, we bring back with us what Bacon, in his nervous language, calls the "*smoke and tarnish of the furnace.*" The mind seems to be broken down to the littleness of the objects which it has been habitually contemplating; and we regard the faculties that measure earth and heaven, and that add infinity to infinity, with a curiosity of no greater interest than that with which we investigate the angles of a crystal, or the fructification of a moss. Such are represented by a philosopher* of high standing, as the inevitable consequences of a too exclusive devotion to the study of mere physical phenomena. But I would advance a step further, and maintain that a knowledge of the philosophy of the human mind is indispensable to the successful prosecution of physical science; that without a knowledge of mental phenomena, a high degree of perfection and accuracy could not be attained in any of the collateral branches of knowledge.

I cannot dismiss this division of my subject without directing your attention to another branch of study intimately associated with the science of medicine and mental philosophy, and one most essential to the education of the psychological physician. I allude to *logic*, or the art of reasoning. Need I advance an argument, to establish the importance of a more general knowledge of that science which analyses the operations of the human understanding in the pursuit of truth. Mr. Stuart Mill places this science upon its right basis, when he argues that logic is not (as some maintain) the science of *belief*, but of *proof* or *evidence*. Its object is not to teach the physician what the symptoms are which indicate disease: these he must acquire from his own experience and observation, or from that of others. But logic, as he maintains, sits in judgment on the sufficiency of that observation and experience to justify his rules, and on the sufficiency of his rules to justify his conduct. It does not give him proofs, but teaches him what makes them proofs, and how he is to judge of them. Logic can never show that the fact A proves the fact B, but it can point out to what conditions all facts must conform, in order that they may prove other facts. "It is," says Mr. Mill, "to use the words of Bacon, the *ars artium*, the science of science itself. All science consists of *data* and of conclusions from these *data*, of proofs and what they prove. Now, logic points out what relations must subsist between data and whatever can be con-

* Browne.

cluded from them; between proof and anything which it can prove." It is not sufficient to establish that a knowledge, a technical knowledge, of the process of reasoning, an apt appreciation of the use and application of recognised logical formulæ, is not actually necessary to enable a person to reason rightly, in order to prove that an acquaintance with the science is not indispensable to the physician. It is true, as Dr. Gregory observes, that a sailor may navigate a ship, who is ignorant of the principles of navigation, and a person may construct a dial, who knows nothing of the principles of astronomy, spherical trigonometry, or the projection of the sphere. Extensive experience, a natural quickness of apprehension, an intuitive perception of the relationship between phenomena, a capability of ready generalization, often make a man a good practical logician who has no knowledge of a syllogism, or of the elements of logical science. Among the higher order of practical intellects there have been many of whom it was remarked, "how admirably they suit their means to an end, without being able to give any sufficient reason for what they do, and apply, or seem to apply, recondite principles which they are wholly unable to state."

But, as medical philosophers, we must not be satisfied with this natural aptitude or intuitive perception of the principles of logic. The science of medicine is especially amenable to the rules of logical and inductive reasoning. Having to unravel the mysterious phenomena of life, the investigation and treatment of those deviations from its normal state, termed disease, peculiarly expose us to many sources of error and fallacy, unless we cautiously keep in view the great truths inculcated by the Baconian philosophy, and are guided by the unerring principles taught by its illustrious founder—

> "The great deliverer, he who from the gloom
> Of cloistered monks, and jargon-teaching schools,
> Led forth the true philosophy."

There are but few gifted men in our profession, or in any other walk of modern science, of whom we could, in justice, say that they were able to dispense with the patient study of facts, or with the recognised *formulæ* of logical and inductive science. It was remarked of the immortal Newton, that he appeared to arrive *per saltum* at a knowledge of principles and conclusions that ordinary mathematicians only reached by a succession of steps, and after the result of much labour, long-continued and profound meditation. It is only by strictly applying the principles of the inductive process of reasoning—by which we conclude that what is true of certain individuals of a class, is true of the whole class; or that which is true at certain times, will be true under similar circumstances at all times—that medicine will take rank with the exact sciences, and its cultivators have a right to claim a foremost position among the distinguished philosophers of the day. In the study of medicine, perhaps more than in any other science, we are peculiarly exposed to the danger of adopting false facts, of being seduced by specious and hasty generalizations, and led into error by deducing general principles from the consideration of a few particulars—the bane of all right and sound reasoning—the foundation of all bad philosophy. It is on this account that logic should form a part of the curriculum of our medical schools.

In analysing the passions, it is our duty to ascertain, if possible, the nature of the mysterious union existing between particular organic tissues and certain emotions of the mind. Why, for example, should the passion of fear specially affect the heart, and, if of long continuance, induce actual physical changes in the centre of the circulation? How can it be explained that in certain diseases of the heart the patient often manifests a morbid apprehension of some approaching calamity? Again, it is for the medical psychologist to ascertain the *modus operandi* of the passion of anger upon the hepatic secretion, and the re-action of disease of the liver upon the irascible temperament. How does fear cause diarrhœa, and predispose the system to the action of contagion? Again, may it not be possible to elucidate the action of terror in suddenly arresting hæmorrhage; and explain why the apprehension of threatened disgrace checks attacks of convulsive disease arising from a morbid principle of imitation, and arrests the progress of epidemic suicide? The emotion of hope is known specifically to influence the respiratory functions, and in the last stages of pulmonary disease the patient is often buoyed up with the certain expectation of recovery, whilst the angel of Death is hovering about him. "How frequently have I seen the delicate female, in the last stage of pulmonary consumption, lighted up, and everything assume a bright and cheerful aspect about her. New schemes of happiness have been contemplated, new dresses prepared, and everything was brilliant in her prospects, whilst her parents lived under the greatest apprehension and solicitude, the physician seeing nothing but inevitable fate for the poor victim whose distemper has deluded her."*

In endeavouring to solve these and other subtle points in psychology, we must be prepared to encounter the ridicule and opposition of those who taboo all such speculations as futile and presumptuous. In our patient and persevering study of abstract philosophical truth, we must not be discouraged by such indifference and opposition. It may be legitimately within the compass of the medico-psychologist, aided by discoveries in physiological and other collateral sciences, to unravel the nature of that mysterious union existing between mind and matter; and to trace the origin and source of the emotions, and the mode in which spirit and matter reciprocally act upon each other. The man devoted to the discovery of these great truths may be compelled to resign himself to the neglect and contumely of his contemporaries. Such, alas! has too often been the fate of those great and noble spirits who have shed undying lustre on the land which gave them birth, and the record of whose deeds forms the brightest spot in our country's annals. It is the recollection of the history of such martyrs to science as Harvey and Jenner, which induces us to exclaim with Coleridge, "Monsters and madmen are canonized, whilst Galileo is buried in a dungeon!" A Brahmin crushed with a stone the microscope that first developed to his vision living things among the vegetables of his daily food. Professor Sedgwick, when referring to this fact, observes, "The spirit of the Brahmin lives in Christendom. The bad principles of our nature are not bounded by *caste* or *climate*, and men are still to be found, who, if not restrained by the wise and humane laws of their country, would try to stifle by personal violence, and crush by brutal force, any truth not hatched by their own conceit, and confined within the narrow fences of their own ignorance."

In analysing the nature of the passions, ascertaining their connexion with each other, mode of action upon the system, and special relationship to certain organic structures, it is necessary to recollect that they are planted in us for wise, beneficent, and noble purposes; and it is only when they are abused, and not

* Sir H. Halford.

subjected to a healthy discipline, that they induce disease, and affect the duration of life. While the impressions made upon the nervous system are moderate, and restrained within due bounds—when there is a natural gratification of the passions, guided and ennobled by reason, the effect produced upon the system is rather of a beneficial than of a pernicious nature. The "passions are, in morals," says Sydney Smith, "what motion is in physics: they create, preserve, and animate; and without them, all would be silence and death. Avarice guides men across the deserts of the ocean; Pride covers the earth with trophies, mausoleums, and pyramids; Love turns men from their savage rudeness; Ambition shakes the very foundation of kingdoms. By the love of glory, weak nations swell into magnitude and strength. Whatever there is of terrible, whatever there is of beautiful in human events, all that shakes the soul to and fro, and is remembered while thought and flesh cling together,—all these have their origin in the passions. As it is only in storms, and when their coming waters are driven up into the air, that we catch a glimpse of the depths of the ocean; so it is only in the season of perturbation that we have a glimpse of the real internal nature of man. It is then only that the might of these eruptions, shaking his frame, dissipate all the feeble coverings of opinion, and rend in pieces that cobweb veil with which fashion hides the feelings of the heart. It is then only that Nature speaks her genuine feelings; and as at the last night of Troy, when Venus illumined the darkness, and Æneas saw the gods themselves at work, so may we, when the blaze of passion is flung upon man's nature, mark in him the signs of a celestial origin, and tremble at the invisible agent of God."

> "Who that would ask a heart to dulness wed,
> The waveless calm, the slumber of the dead?"
>
> CAMPBELL.

Having, I trust, established the necessity of a more general acquaintance with mental philosophy, it is now my province to demonstrate its practical application as a therapeutic agent in the hands of the physician.

From the annals of empiricism the psychological physician may glean many useful lessons. "*Fas est et ab hoste doceri,*" is a maxim as applicable to medical as it is to moral and political science. May not the success that sometimes follows the administration of an extravagantly eulogized nostrum often depend upon the *moral confidence* inspired in its much-vaunted efficacy? Medicine often has a curative efficacy *because the patient is told and believes that it will cure*—is unerring in its effects—infallible in its results. Let us learn a lesson from this fact, and remember the observation of Coleridge, that "he is the best physician who is the most ingenious inspirer of hope." How often has a disease which has baffled the skill of the scientific, practical man, vanished before the spell of a village witch. A patient afflicted with a malady which refused to yield to the demands of legitimate medicine, surrendered himself into the hands of a notorious quack. A friend endeavoured to rescue him from the grasp of the charlatan. He saw the daily fee accompanying the daily deceit, and expostulated; when the patient exclaimed, "For God's sake, destroy not the hopes that man holds out to me: upon them I live! without them I die!"

In acute attacks of disease, the patient who has the least fear of dying has, *cæteris paribus*, the fairest prospect of recovery. The tonic and often stimulating influence of HOPE not only arrests the progress of organic mischief, but invigorates the system, thus warding off the approach of disease. Aretæus, appreciating the importance of rousing and supporting, by means of moral agents, the nervous system, when in a state of depression and debility, expressly counsels the patient to be of good heart, and advises the physician to entertain him with agreeable conversation, and to do his utmost to encourage hope and confidence. With a view of abstracting the mind of the patient from a contemplation of his own sufferings, he directs that his mind should be diverted with the sight of plants in full bloom, and agreeable paintings; and suggests that the bed of the patient should be placed near a window commanding a beautiful prospect. The chamber, he says, should be strewed with flowers; amusing books should be read, and the soothing influences of music should be brought to bear upon the moral treatment of the case. The mind of the patient should not be permitted to dwell upon his physical malady; and he should be constantly buoyed up with the hope of recovery. When speaking of the plague of Athens, Thucydides says that "the most affecting circumstance connected with the epidemic was the great and fearful mental dejection which accompanied the attack. The mind appeared at once to sink into despair, and the patient often gave himself up without a struggle."

We all fully appreciate the potency of mental depression among the predisposing causes of contagious disease. During the prevalence of epidemic diseases, it may be a matter worthy of consideration whether there are not some powerful MORAL REMEDIES, by means of which they may be shorn of much of their virulence. It is a question entitled to serious discussion, what are the best means within our reach to effect so desirable an object? Many may smile at the idea of attempting, by any mental measures, to create a revulsion in the public mind, and thus to destroy, if possible, all fear and apprehension. When Rome was threatened with pestilence, the public authorities marched in solemn procession to the national temple, and means were adopted for appeasing the anger of the gods. The psychological effect of this, to our minds, superstitious proceeding, was to allay public apprehension, and to excite hope and confidence. May not we adopt measures somewhat analogous to arrive at similar results? Have we not within our power effectual means of acting upon the public mind *en masse*, for creating, during the existence of those fearful panics which so often accompany the prevalence of pestilential diseases, a new turn to the current of thought, and of dispelling unnecessary fears and morbid apprehensions? God has so intimately associated the spiritual with the material portion of our organization, that He will not consider that we are slighting His dispensations, or making light of His awful providence, if, in obedience to His will, and in conformity to the recognised laws influencing the mysterious union of mind and matter, we adopt moral or mental means for curing or preventing disease.

Such being a view of the question sanctioned by Religion and Science, it behoves us to consider whether some measures might not be adopted for the purpose of abstracting the public mind from its own depressing apprehensions, thus rendering the system less liable to be acted upon by those physical agents alleged to give origin to the disease. This is only suggestive; it may be entirely impracticable; but whether it be so or no, I have not the slightest doubt of the soundness of the *principle*, and of the importance of adopting every legitimate means of allaying any panic that may occur, and of looking beyond the mere physical means at our disposal, for the prevention and cure of disease.

It is our duty, during these fearful epochs, to dismiss from the mind the contemplation of subjects calculated to awaken gloomy apprehensions, to depress the feelings, and exhaust the nervous energy. Every reasonable mode of inducing cheerfulness and serenity should be encouraged. Constant and agreeable occupation will do much good. An effort should be made to excite emotions of a pleasurable character. The exercise of charitable feelings, the determination to keep in abeyance all the corroding passions, such as anger, jealousy, revenge, covetousness, and the effort to cultivate "love, peace, and good-will toward men," will be found of positive advantage in invigorating the *physique*, and thus rendering innocuous the poison of contagion.

We should never forget that those whose vital powers are debilitated are the most susceptible to epidemic maladies,—that the depressing emotions induce this predisposition more certainly than any other cause. A humble reliance on the will of God, a well-sustained piety and cheerfulness, are the safest and most legitimate means (apart from the use of physical agents) of preventing the spread of epidemic maladies. During the prevalence of any such visitation, it is our duty individually, as well as nationally, to fortify and strengthen the system, by resolutely determining not to yield to useless fears and childish apprehensions; and, so far as it is in our power, to inspire ourselves and our neighbours with energy and courage, and, as a powerful prophylactic agent, to cultivate

"Sweet, unanxious quiet for the mind."

They are the happiest, the healthiest, and the longest-lived, who systematically cultivate *ease of mind*. On this subject a popular writer has justly observed, that "This happy state of mind is in a great measure within the reach of all who diligently seek after it. It does not depend upon the amount of our worldly possessions, but upon our mode of using them not upon our ability to gratify our desires, but upon our regulation of them. They who diligently cultivate the habits necessary to attain ease of mind, place themselves almost above its disturbance. To the mortifications of disappointed ambition they are not at all exposed, and by the crosses of adverse fortune very little; whilst unavoidable afflictions in the well-constituted soften rather than sour the mind, and cannot be said to destroy its ease. Like cypresses, they throw a shade over the current, but in no way disturb its smoothness. Strict and constant discipline can insure ease of mind in poverty and privation."*

To the physician specially occupied in the investigation of the deranged conditions of the mind, how indispensable is a knowledge of mental philosophy! Unless acquainted with the constitution of the human understanding, we are but ill adapted to unravel or appreciate the intricate phenomena of its disordered action, or to apply for their cure or alleviation those medical and moral agents which advanced science has placed within our reach. Before the morbid mental state can be diagnosed or understood, it is necessary for the physician to be intimately conversant with some normal standard of comparison, otherwise he possesses no test by means of which he can arrive at a safe conclusion. Who would confide in the judgment of a physician who endeavoured, by means of the stethoscope, to ascertain the nature of any particular disease of the heart and lungs, if he were ignorant of the normal action of those organs! Is not a knowledge of the healthy operations of thought as necessary to the physician who is called upon to pronounce whether, in any given case, an apparently suspicious deviation from the normal condition of the intellect is or is not the effect of disease?

Is it necessary that I should seriously endeavour to establish the importance, not only of a knowledge, but of a profound knowledge, of the human understanding, its affections, propensities, emotions, and its instincts, to those occupied in the study of mental alienation, and specially and personally engaged in the treatment of the insane? Alas! an acquaintance with this section of philosophy is rarely deemed necessary in the education of those intended for psychological practice. Hitherto, with few exceptions, those engaged in this branch of practical medicine have not only been grossly ignorant of the constitution of the human mind, but unacquainted with the first principles of medical science. It is only in recent times that it has been considered necessary to ascertain whether parties desiring to undertake the care and treatment of the insane† have any other qualification to

* "The Original," by Walker.

† When asylums for the insane are entrusted exclusively to physicians acquainted with the *anatomy of the human mind*, or, in other words, with the science of *medical psychology*, they will realize the conception of the great Esquirol, and become "instruments of CURE, and, in the hands of the skilful physician, most powerful therapeutic agents against mental maladies."

recommend them to the office than the possession of a house of certain dimensions, and a sufficient amount of capital to enable them to carry into effect the mere commercial speculation. A surgeon, before he undertakes the performance of an operation, or the treatment of a serious, or even of a trifling injury, is expected to have devoted many years to the study of anatomy and the practice of surgery. A physician is considered to be conversant with the different branches of medical science; to have watched by the bedside the operation of various medicinal agents, exhibited in certain states of disease; and to be thoroughly conversant with the science of therapeutics and the phenomena of morbid action. But a knowledge of the most intricate, complicated, and subtle phenomena with which we have to deal—namely, the faculties of the human mind—is considered to come by intuition, no preparatory psychological education being deemed necessary to those devoted to the treatment of the insane. What would be said of our own mental condition, if we were to place in the hands of a blacksmith a delicate chronometer, for the purpose of having its movements regulated? And should we not expose ourselves to severe animadversion, if we permitted a man ignorant of the anatomical construction of the body, to cut down upon the subclavian artery, for the purpose of applying a ligature to the vessel? Is it less absurd, less destructive to the integrity of the intellectual part of our constitution, to place under the care of persons grossly ignorant of the elements of the science of mind, cases of disease requiring, above all others, for their judicious and successful management, an intimate knowledge of healthy mental phenomena? "Great powers of reason are requisite," says Vogel, "to understand men destitute of reason." To treat the various phases of disordered mind with any hope of a successful issue, requires on the part of the psychological physician qualities of mind rarely seen in combination—tact, presence of mind, judgment, a ready appreciation of intricate morbid mental phenomena, a delicacy of taste, a high *morale*, a steadiness of purpose, elevation of character, great command of temper, and volitional power and resolute determination not to allow any amount of provocation to interfere with that calmness and serenity so indispensably necessary on the part of those brought into immediate association with the insane. If the mind be the instrument upon which we are to operate in carrying out any systematic plan of moral treatment—if it be the duty of the physician to perseveringly "combat with delusions and hallucinations, and to substitute for them correct and healthy impressions; to strengthen these impressions by judicious and repeated repetitions; remove perverted trains of reasoning—replace them by correct induction, and give them the power and influence of habit and frequent association:" how, I ask, can he make any progress in this mode of treatment so long as he is ignorant of the *material* with which he is to work—in fact, with the faculties of the human understanding? If the man who has the advantage of an ordinary medical education is, on account of his ignorance of the philosophy of mind, obviously unfitted for the serious duties of treating its disorders scientifically and successfully, what language can convey our impression of the folly, the barbarity and heartlessness, of entrusting the management of the insane to those who are not members of the profession at all, and who have enjoyed no more psychological or general education than that derived from their having acted as attendants in asylums, or that which they have received at a village school? Need we feel surprise at the little advancement made in the science of cerebral pathology, and the amount of public odium which has, alas! for so many years attached to those specially engaged in this anxious and important branch of practice, when we consider into whose hands this class has unhappily fallen? I trust, however, the day is not very remote, when the psychological physician, engaged in the treatment of insanity, will take his proper and legitimate position in the ranks of honourable and scientific men; and the opprobrious epithet with which the vulgar and illiterate assail him will be expunged from the vocabulary. When that epoch arrives, the public and the profession will esteem, respect, and venerate those who, at great and heroic personal sacrifices (often of health, life, and reason), devote their acquirements, energies, and talents, for the benefit of this section of the afflicted family of man. "I am at length rewarded," says Müller,* "since after twenty-six years' intercourse with the insane, I have not become insane myself." In a letter to Pinel it is observed, "The labourer in lead-works is thankful if he escapes lameness, and the medical attendant of a madhouse, if he does not there leave his reason. A more deliberate sacrifice to the mightiest good of mankind is not conceivable."†

* Physician to the Julius Hospital, Wurzburg.
† "Aspects of Medical Life," by Dr. Mackness.

There is another practical point connected with the study of medical psychology, which comes within the range of our investigation. It has reference to the influence of the will upon the physical organism. It has been maintained that the persistent direction of the volitional power to a particular organ or structure will eventually induce a morbid activity in the part, and give rise to lesions in the organic tissue itself. In many cases of hypochondriasis, a disease often associated with some form of visceral derangement, I have no doubt the sufferings, both mental and physical, are often aggravated by the patient imagining some particular structure or viscus to be the seat of disease; and from that circumstance, the attention being constantly directed to the organ, actual molecular changes in the organic elements of the part are induced. The persistent current of mental impulse, emotion, or volition towards an organ, impels to it an amount of nervous energy and blood sufficient to derange the circulation, and thus interfere with the function of nutrition, and induce organic alterations in the tissue. Does this fact admit of a psychical explanation. Viewing practically the operations of volition, I would ask whether it be not possible to prevent or cure actual physical and mental disease by an effort of the will; and if so, what is the rationale of the process? The will, by a constant exercise of its powers, has been known to acquire an influence over the involuntary organs. The case of Colonel Townsend is familiar to us all. This gentleman, by an effort of the will, could easily suspend the action of the heart, and thus induce, for a period, all the symptoms of apparent death. Celsus refers to a priest who exercised the same power over all the vital functions. In the language of Burton, "he could separate himself from his senses when he list, and lie like a dead man, void of life and sense."* Great expecta-

* A Colonel Townsend, residing at Bath, sent for Drs. Bayard and Cheyne, and a Mr. Skrine, to give them some account of an odd sensation, which he had for some time felt, which was, that he could expire when he pleased, and by an effort come to life again. He insisted so much on their seeing the trial made, that they were forced at last to comply. They all three felt his pulse, which was distinct, and had the usual beat. He then composed himself on his back for some time. By the nicest scrutiny, they were unable to discover the least sign of life, and at last were satisfied that he was actually dead; and were just about to leave him, with the idea that the experiment had been carried too far, when they observed a slight motion in the body, and the pulsations of the heart returned, and he quite recovered. In the evening of the same day, however, he composed himself in the same manner, and really died.

tions may be entertained of recovery from an attack of illness, if the patient, with a recognition of his duty of submission to the will of God, resolutely determines not unnecessarily to yield to physical disease. The determination to be well, will, in certain morbid states of the system, do much to facilitate recovery, and will materially aid the physician in the exhibition of his curative agents. The author of *The Original* relates a curious circumstance connected with his own bodily health, which illustrates the power which the mind exercises over physical disease. He says: "Some months before I was born, my mother lost a favourite child by illness, owing, as she accused herself, to her own temporary absence; and that circumstance preyed upon her spirits and affected her health to such a degree, that I was brought into the world in a very weakly and wretched state. It was supposed I could not survive long, and nothing, I believe, but the greatest maternal care and tenderness preserved my life. During childhood, I was very frequently and seriously ill, often thought to be dying, and once pronounced to be dead. I was ten years old before it was judged safe to trust me from home at all, and my father's wishes to place me at a public school were uniformly opposed by my various medical advisers, on the ground that it would be my certain destruction. Besides continued bilious and inflammatory attacks for several years, I was grievously troubled with an affection of the trachea; and many times, after any excess in diet or exertion, or in particular states of the weather, or where there was new hay or decayed timber, my difficulty of breathing was so great, that life was miserable to me. On one occasion, at Cambridge, I was obliged to send for a surgeon in the middle of the night, and he told me, the next morning, that he thought I should have died before he could have opened a vein. I well recollect the relief it afforded my agony, and I only recovered by living for six weeks in a rigidly abstemious and most careful manner. During these years, and for a long time after, I felt no security of my health. At last, one day, when I had shut myself up in the country, and was reading Cicero's treatise *De Oratore*, some passage, I forget which, suggested to me the expediency of making the improvement of my health my study. I rose from my book, stood bolt upright, and determined to be well."* Mr. Walker then proceeds to narrate, in a number of

* "The Original," by Walker.

amusing essays, how he carried his resolution into effect. The result was a complete restoration to health, which he enjoyed until a short period previous to his death.

For the cure of many of the disorders of the nervous system, it is often necessary for "the mind to minister to itself." If the patient confess an inability, in the more advanced stages of mental disease, by an effort of volition to "pluck from the memory a rooted sorrow," he undoubtedly has the power in the earlier or incipient forms of disordered mind, to destroy, by a resolute effort of the will, "those false creations of the heat-oppressed brain," those "thick-coming fancies," and those irregularities of thought and conduct, which, if permitted to run riot and uncontrolled, would induce the more serious, dangerous, and perhaps incurable forms of mental derangement. "By endeavouring, from benevolent motives, to smother the expression of our sorrows," says Dr. Reid, "we often mitigate their inward force. If we cannot imbibe the spirit, it is often profitable, as well as good-natured hypocrisy, to put on the appearance of cheerfulness."

"By *seeming* gay, we grow to what we seem."

Let us, as psychological physicians, impress upon the minds of those predisposed to attacks of mental aberration and other forms of nervous disease, the important truth, that they have it in their power to crush, by determined, persevering, and continuous acts of volition, the "floating atoms, the minute embryos, the early scintillations" of insanity. Many of the diseases of the mind, in their premonitory stage, admit, under certain favourable conditions, of an easy cure, if the mind has in early life been accustomed to habits of self-control, and the patient is happily gifted with strong *volitional power*, and brings it to bear upon the "scarcely-formed filaments of mental disease." "We should have fewer disorders of the mind if we could acquire more power of volition, and endeavour, by our energy, to disperse the clouds which occasionally arise within our own horizon; if we resolutely tore the first threads of the net which gloom and ill-humour may cast around us, and made an effort to drive away the melancholy images of the imagination by incessant occupation."*

It is sometimes necessary, in the application of moral influences,

* Essays on Hypochondriasis, &c., by Dr. Reid. (German Edition.)

to rouse the apprehensions of our patient by pointing out to him his position as an accountable agent. I cannot better illustrate this psychological function of the physician than by quoting an anecdote which has been recorded of the late Mr. Abernethy. A patient was brought to St. Bartholomew's Hospital with strangulated hernia. As the symptoms became alarming, the propriety of an operation was suggested to him, but he resolutely refused compliance; and although his alarming situation was fully pointed out, he persisted in his determination. On the following day a consultation was held, and it was agreed that no alternative remained but a speedy death, unless the operation were performed. When this was announced to the sufferer, he exclaimed, "I will rather die than submit!" As the surgeon and pupils were leaving the hospital, Mr. Abernethy entered. The position of the patient was at once explained to him. He immediately went to the bedside of the man, when the following conversation ensued:—"Well, well, my good fellow?" said Abernethy.—"They want," replied the patient, "to persuade me to be operated upon; but I would rather die!"—"Well," rejoined Mr. Abernethy, "I am sorry the operation is necessary; but have you thought of what there is after death? There is a day of judgment, and you will and must give an account of yourself to God. God has placed within our hands the means to use, and we must use them. If you refuse to use the means God has thus given, and which we think may save your life, you are, in a measure, answerable for your own death, and must account to God for this, with your other sins." The man appeared much impressed with Mr. Abernethy's appeal, and for a period continued silent and in deep thought. Mr. Abernethy said, "I will leave you for a few minutes to consider the subject." On returning, the man exclaimed, with great eagerness and decision, "I will submit to any operation that is necessary!" The operation was immediately performed, and his life was preserved.*

I have not yet spoken of the conduct of the physician whose special duty it is to attend and officiate at the period of parturition. There are no occasions when it is so essentially necessary for the medical practitioner to zealously watch the operation of moral causes upon the *physique* of his patient. The successful progress of labour is often dependent upon the temperament of

* Dr. Cooke.

the physician. The patient, anxious about her own state, and nervous as to the issue, watches every movement of the physician —his very attitude—his every look—his walk; his remarks, either addressed to herself, or those in the room, are closely scanned, and have a beneficial or a disastrous influence upon the mind of the patient. In proportion as the obstetric physician recognises the potency of mental agents upon the mind of the patient, and is facile in their adaptation to the idiosyncrasy of those with whom he is brought into contact, will he be successful in the practice of this important section of medical science.

The physician is often called upon, in the exercise of his responsible vocation, to discharge medico-theological functions. It is occasionally our painful duty to sit by the couch of the dying, and to witness the last fatal conflict between mind and matter. It is on such occasions that we have, either in co-operation with the recognised minister, or in his temporary absence, an opportunity of whispering words of comfort and consolation to the wounded spirit, and of directing the attention of the patient, and those immediately about him, to the only true and legitimate source of the Christian's hope. Let us not lightly esteem or neglect the solemn functions thus imposed upon us. It may be our privilege to co-operate with those whose sacred duty it is to inculcate the precepts of our holy religion, and to suggest, without subjecting ourselves to the imputation of officiousness, the *degree* and *kind* of conversation admissible under certain physical or mental states. A zealous but indiscreet clergyman may, by the character of his admonitions, fatally interfere with the successful progress of an acute case of disease, and inadvertently produce an amount of mental and physical depression, from which the patient may never rally. In the exercise of this serious, this important, and imperative duty, the object should be to soothe, not to distract, the mind; to elevate, not to depress, the emotions; to inspire a holy reverence and simple reliance upon that DIVINE BEING who is the FOUNTAIN OF ALL JUSTICE, and the RESERVOIR OF ALL MERCY. Our Saviour should be represented, not as the God of terror, but as a God of LOVE and MERCY. "What painter who has sketched the portrait of our Saviour, ever thought of arming him with thunder? No: love was HIS weapon; and this is the weapon his ministers should chiefly employ."*

* "The Velvet Cushion," by the Rev. J. W. Cunningham.

"Thou, fair Religion, wast designed,
Duteous daughter of the skies,
To warm and cheer the human mind,
To make men happy, good, and wise,
To point where sits, in love arrayed,
Attentive to each suppliant call,
The God of universal aid—
The God, the Father of us all."
PENROSE.

The physician, whilst officiating under these painful circumstances, may have it in his power to disarm the imagination of the dying, of those unphilosophical, phantasmal, and often superstitious notions with which the morbidly active fancy occasionally invests the act of death itself. Is the fear of death a natural and healthy feeling? Many eminent divines entertain this idea. The instincts of our nature, however, recoil from the thought of dissolution; the soul "shrinks back upon herself" at the idea of annihilation and destruction. This horror of death prevailed to a great extent in ancient times, particularly among the Jews. The weeping and wailing referred to in Scripture (Mark. v. 38) may "be traced to an early tradition among them that an evil spirit, whom they called the 'angel of death,' had special permission to torment persons in their dying hour, and even long after their decease. This angel they represented as standing over the sick man with a drawn sword, then distilling into some part of his body the poisonous death-drop, and afterwards going to sit upon his grave, to terrify his unresting spirit with sounds and sights of woe."*

Hazlitt imagines that the subject of death is made ghastly to the imagination, by our associating with it the idea of life. We think how WE should feel, not how the DEAD feel.

"Still from the tomb the voice of nature cries,
Even in our ashes live their wonted fires."

"The melancholic appearance of a lifeless corpse, the mansion provided for it to inhabit, dark, close, and solitary, are shocking to the fancy, but it is to the fancy only, not to the understanding; for whoever consults this faculty, will see at the first glance that there is nothing dismal in all these circumstances. If the corpse were kept wrapped up in a warm bed, with a roasting fire in the chamber, it would feel no comfortable warmth therefrom. Were stores of tapers lighted as soon as day sets in, it would see no object

* "Christian Consolation," by the Rev. D. Moore, M.A. 1848.

to divert it. Were it left at large, it would have no liberty, nor if surrounded by company would it be cheered thereby; neither are the distorted features depressions of pain, uneasiness, or distress. This every one knows, and will readily allow, upon being suggested, yet still cannot behold, nor even cast a thought upon these objects without shuddering; for knowing that a living person must suffer grievously under such appearances, they become habitually formidable to the mind, and strike a mechanical horror, which is increased by the customs of the world around us."*

But if such apprehensions of death haunt and distress the imagination of those eminent for their piety, great natural sagacity, and for their high order of intelligence, they are far from having a general influence. I quite concur in the sentiments expressed by the late Sir Henry Halford, who, when referring to the calmness, serenity, and Christian resignation exhibited by many at the awful moment of death, says, "Of the great number to whom it has been my painful professional duty to have administered in the last hour of their lives, I have sometimes felt surprised that so few have felt reluctant to go to the

'——————undiscovered country,
From whose bourne no traveller returns.'

Many, we may easily imagine, have manifested their willingness to die, from an impatience of suffering, or from that passive indifference which is sometimes the result of debility, and extreme bodily exhaustion. But I have seen those who have arrived at a fearless contemplation of the future, from faith in the doctrines which our holy religion teaches; such men were not only calm and collected, but even cheerful, at the hour of death; and I never quitted such a sick chamber without a wish that my last end might be like theirs. Some, indeed, have clung to life anxiously—painfully; but they were not influenced so much by the love of life for its own sake, as by the distressing prospect of leaving children dependent upon them to the mercy of the world, deprived of their parental care; in the pathetic language of Andromache—

'Νῦν δ'αν πολλά παθησι, φιλον ἀπὸ Πατρὸς ἀμαρτων.'

These indeed have sometimes wrung my heart."†

* Tucker.

† Sir H. Halford's Essays and Orations.

Cicero is said to have complained that the fear of death hung over him like the stone of Tantalus. All his philosophy and extraordinary intellectual power did not preserve him from childish apprehensions of death. "*Mors, quæ quasi saxum Tantalo, semper impendet.*"*

Dr. Johnson had always an intense dread of death, even when in the enjoyment of perfect health. He says in one of his letters to Boswell, "I cannot think without emotion of the removal of any one I know from one state to another." In a letter to Dr. Taylor, he exclaims, "O my friend, the approach of death is very dreadful! I am afraid to think of that which I cannot avoid!" He told Dr. Hawkins that he never had a moment in which death was not terrible to him. He died eventually of dropsy. In order to prolong his life, he procured a lancet, with which he was going to puncture his legs, which were much swollen. He was, however, prevented from doing so; and when he was entreated not to do so rash an action, he said that he would not. Shortly afterwards his arm was seen to be moving under the bed-clothes, and upon turning down the clothes, his friends found that he had been plunging a pair of scissors into the calf of each leg. Upon being expostulated with, Dr. Johnson feelingly exclaimed, "I want length of life!—length of life!"

It may be our duty to explain to those labouring under mortal disease, with certain dissolution in immediate prospect, and who express, with what may be termed some degree of truth, a morbid apprehension of the fatal issue, that, reasoning from analogous phenomena, we are not justified in believing that the act of death is accompanied with any physical agony. The violent muscular convulsions simulating epilepsy, which occasionally accompany the act of dying, naturally suggest to the vivid imagination of the bystander the idea of intense suffering. It is within the range of our legitimate function to expose this fallacy, by explaining, that although the patient may apparently suffer much a short period before death, the act of dying cannot, reasoning physiologically, be painful, *consciousness* being then entirely suspended. Dr. Symonds observes that "the practitioner ought to be able to console the friends of the dying, by the assurance

* Cic. Tusc. Desp., v. 40.

that, whatever may have been the previous torture, it must be all over when once those changes begin in which death essentially consists. He must explain to them how, upon the failure of the circulation, the functions of the brain must cease by necessity; that if the cessation of the former be gradual, that of the latter may, and often does, precede it; that if the mortal process begins in the lungs, unconsciousness precedes the want of circulation; and if in the brain, that an injury of this organ, sufficient to affect the lungs and the heart fatally, is sure to annihilate its own sensibility. The muscular spasms, the slow, gasping, and gurgling breathing, the collapsed and distorted features, though in some cases accompanied by feeling, are altogether independent of it. Convulsion is not, as superficial observers often interpret it, the sign of pain, or the result of an instinctive effort of nature to get rid of the cause of pain—it is an affection of the motific, not the sensific part of the nervous system. The pangs of disease may last till within a short period of death, but it is a great error to attribute them to the process that brings them to an end. Such cases are rare; it is far more common for the sensibility to be blunted, or for the cause of pain to subside before the phenomena of dying commence." *

I will not be guilty of the presumption of attempting to draw aside the veil which conceals from mortal vision the condition of the spirit whilst traversing "these painful passages."† In vain have the most highly-gifted minds, the most exalted imaginations, and the most sublime flights of poetry, endeavoured to convey to our understanding a conception of the state of the soul during that terrible conflict which holy men have taught us to believe takes place in the act of death. To our finite conceptions the struggle is ended as soon as life appears to be extinct. Is such the fact? or is the process of dying still going on, as some have supposed, even after the heart has ceased to pulsate?‡ These subtle and

* Art. "Death." Cycl. Anat. and Phys. † Milton.

‡ "More than a hundred experiments on living animals have satisfied me," says Richerand, "that the intestines are always the last part in which the traces of life may be discovered. Whatever may be the sort of death by which they are destroyed, peristaltic motions are still continued in this canal, while the heart has already ceased to beat, and the rest of the body is all an inanimate mass." Dr. Smith observes that the corollary from this position is obviously the propriety of applying stimulants to the intestinal tube, in cases of suspended animation.

mysterious questions are, I fear, beyond the range of the most acute and deeply-thinking philosophers.*

To many minds the subject of death presents great attractions. Its awful sublimity, the mysteries that hang over it, its natural associations with all that is tender and pathetic, invest it with a poetic charm to which it is impossible for a man of taste, intellect, and feeling to be insensible.

"Thoughts unspeakable
Crowd in my heart to burning, when I hear
Of this almighty death, who is, it seems,
Inevitable. * * * *
* * * * * * *
It hath no shape, but will absorb all things
That bear the form of earth-born being.
I knew not that, yet thought it, since I heard
Of death; although I know not what it is,
Yet it seems horrible. I have looked out
In the vast desolate night in search of him,
And when I saw gigantic shadows in
The umbrage of the walls of Eden, chequered
By the far-flashing of the cherub's sword,
I watched for what I thought his coming, for
Dark fear rose longing in my heart to know
What 'twas that shook us all—but nothing came;
And then I turned my weary eyes from off
Our native and forbidden paradise,
Up to the lights above us in the azure,
Which are so beautiful."†

What more sublime than the transition of the soul from one state of being to another! What more mysterious than the passage of the disembodied spirit through the valley of the shadow of death! Who can imagine the feelings of the traveller, or portray to our imaginations the visions of the place?

Viewing, however, the subject before us in a more practical light, and referring to the conduct of the physician at that solemn crisis, I would suggest whether he may not have occasion to point out the propriety of some member of the family being by the bedside of the patient in his last moments, as the approximation of those nearly related to the dying is supposed, upon good grounds, to comfort and sustain the mind, and smoothe the passage to the tomb, although there is no apparent recognition

* Under the heathen mythology, it was believed that the struggles of death continued till Proserpine had cropped the hair on the crown of the head, as victims were treated at the altar. Virgil has preserved this opinion in the fourth book of the Æneid, where he offers so fine a picture of the dying agonies of Dido.

† Byron.

or evidence of consciousness remaining. When Louis XIV. was dying, he turned to his physician, and exclaimed, "It is not so difficult to die as I expected!" Voltaire, in referring to this circumstance, remarked, "All men die with composure and fortitude *who die in company.*" He imagines that the courage of soldiers in the heat of battle is in a great measure owing to the fact of their being surrounded by those who may, in case they should fall, bear testimony to their gallantry and courage. By parity of reasoning, and from the observation of himself and others, he concludes that the actual contact of a relative with the dying man, at the moment of the last struggle, sustains and supports him in the terrible convulsions that ensue, when spirit becomes disembodied from matter.

It is often the painful duty of the physician to intimate to his patient that the last gleam of hope has faded from his mind, and that he must prepare for the painful change which awaits us all. I would impress upon your minds, recognising the powerful influence of depressing mental emotions upon the shattered physical condition, the great importance of not prematurely snatching from under the patient the only prop—frail and fragile as it may be—upon which his and our hopes of recovery rest. To inform a man that he must prepare for death; that his hours are numbered; to bring about his bed the wailing of deep distress, when reasonable expectations exist of his ultimate recovery, would, in certain temperaments, induce the prophetic result.

But an occasion may present itself when it will become our solemn duty to awaken the patient to a sense of his dangerous state and hopeless condition, and to point out to his relatives the necessity of his performing the last sad offices of life. On this subject can I do better than quote the subjoined admirable suggestions?—

"And here you will now forgive me, perhaps, if I presume to state what appears to me to be the conduct proper to be observed by a physician in withholding or making his patient acquainted with his opinions of the probable issue of a malady manifesting mortal symptoms. I own I think it my first duty to protract life by all possible means, and to interpose myself between him and everything that can aggravate his danger; and unless I shall have found him averse from doing what was necessary in aid of my remedies, from a want of a proper sense of his perilous situation,

I forbear to step out of the bounds of my province in order to offer any advice which is not necessary to promote his cure. At the same time, I think it indispensable to let his friends know the danger of his case the instant I discover it. An arrangement of his worldly affairs, in which the comfort or unhappiness of those who are to come after him is involved, may be necessary; and a suggestion of his danger, by which the accomplishment of this object is to be obtained, naturally induces a contemplation of his more important spiritual concerns, a careful review of his past life, and such sincere sorrow and contrition for what he has done amiss, as justifies our humble hope of his pardon and acceptance hereafter. If friends can do their good offices at a proper time, and under the suggestions of a physician, it is far better that they should undertake them than the medical adviser. They do so without destroying his hopes, for the patient will still believe that he has an appeal to his physician beyond their fears; whereas, if the physician lay open his danger to him, however deliberately he may do this, he runs a risk of appearing to pronounce a sentence of condemnation to death, against which there is no appeal, *no hope;* and, *on that account*, what is most awful to think of, perhaps the sick man's repentance may be less available.

"But friends may be absent, and nobody near the patient in his extremity, of sufficient influence or pretension to inform him of his dangerous condition. And surely it is lamentable to think that any human being should leave the world unprepared to meet his Creator and his Judge, 'with all his crimes broad blown!' Rather than so, I have departed from my strict professional duty, and have done that which I would have done to myself, and have apprised my patient of the great change he was about to undergo.

"In short, no rule not to be infringed sometimes can be laid down on this subject. Every case requires its own considerations; but you may be assured that if good sense and good feeling be not wanting, no difficulty can occur which you will not be able to surmount with satisfaction to your patient, his friends, and yourself."*

Apart entirely from the great importance of our having a right appreciation of our position as accountable agents, the cultivation

* Essays and Orations, delivered before the Royal College of Physicians, by Sir H. Halford, Bart., M.D.

of a simple faith in the principles and truths of our holy and revealed religion, during the hours of serious illness, as well as at the solemn moment of death, has, if judiciously regulated, undoubtedly a valuable therapeutic influence.

The serenity, tranquillity, and resignation of the truly Christian mind, in moments of danger and during attacks of acute disease, will often do more to sustain the *vis vitæ*, allay unnatural excitement, and facilitate recovery, than any physical stimuli we may administer. Lord Bacon suggests to the physician that it is a part of his art to smoothe the passage to the tomb, and to render the transition from life to death easy, placid, and gentle. An occasion may present itself, affording to the physician an opportunity of relieving the mind of the dying of oppressing and distressing thoughts that may be interfering with that composure and calmness so necessary and indispensable at this solemn and awful moment. When Goldsmith was upon his death-bed, the intelligent and sagacious eye of his physician recognised that the poet's mind was evidently under the influence of some concealed painful emotions. "I perceive," said his physician to Goldsmith, "that your mind is ill at ease." The poet readily admitted that such was the fact. He was induced to unburden his thoughts; and impressions which would (in all probability) have rendered his last moments miserable, were at once removed by the judicious advice, promises, and consolations of his kind and benevolent physician.

Dr. Armstrong, who had a keen appreciation of the importance of watching the state of the mind during illness, advised Dr. Boot, a few hours before he died, to be always cheerful in his intercourse with the sick; he assured him that the physician may have the power of taking a load from the heart, and infusing into it hope and consolation. Dr. Nichols says, that whatever a man's distemper was, he would not attend him as a physician if his mind were not at ease, for he believed no medicine would have any influence under these circumstances. He once attended a man in trade, upon whom he found none of the medicine he prescribed have any effect. He asked his wife privately whether her husband had not been exposed to some losses in trade? She said "No." He continued to attend him, but no impression could be made on his malady. At length, the man's wife told the physician that she had discovered accidentally that her husband's mind was much troubled by his pecuniary difficulties.*

It will be our province, as psychologists, to trace the connexion between a total want of sensibility in regard to those impressions which affect the eternal welfare of man, and certain morbid conditions of the bodily functions which are generally admitted to exercise an influence over the devotional emotions. Whilst expressing my firm belief in the possibility of a direct interposition of Divine agency upon the mind, inducing spiritual changes in the hearts of those happily brought within the sphere of such holy inspirations, I nevertheless consider it my duty to suggest, that as God, in His great wisdom, often accomplishes His wise designs through the instrumentality of secondary physical agents, it is legitimately within our power, *by watching the state of our mental and physical condition, to adapt the mind for the more ready reception and recognition of those truths the right appreciation of which is so essential to the eternal welfare of the human race.* I would speak with great reverence and caution, and with extreme diffidence, upon subjects so solemn and sacred; yet I would ask, can the physician neglect their philosophical consideration? When alluding to this subject, Baxter, who cannot for a moment be supposed to entertain an irreverent thought in connexion with the subject of religion, observes:—"The want of consolation in the soul is often owing to *bodily disease.* It is not more surprising for a conscientious man, under the influence of a morbid melancholy, to doubt and despair, than it is for a sick man to groan, or a child to cry when it is chastised. Without the physician, in these cases, the labour of the divine would be in vain. Fear may silence the groans of the wounded spirit, but you cannot administer comfort. The consciousness of sin, and the apprehension of the wrath of God, are often the results of bodily distemper."† "There are some cases when a man's thoughts are in a manner forced upon him, *from the present temper and indisposition of his body;* so that, so long as that habit of body lasts, he cannot avoid that sort of thoughts. This is the case of some deeply hypochondriac persons, many of whom will be haunted with a set of thoughts and fancies that they can by no means get rid of, though they desire it never so earnestly

* "De Anima Medica." † "Saints' Rest."

We may properly call these *fancies of their waking dreams*, as their dreams are their sleeping fancies."

"Though we cannot, in many cases, think always of what we would,—nay, though we cannot hinder abundance of thoughts from coming into our minds, *against our will*,—yet it is always in our power to assent to our thoughts, or to deny our consent to them: if we do not consent to them, so soon as we are aware of them, there is no harm done. Should we be haunted with blasphemous thoughts, and cannot get rid of them, we must consider that our thoughts are no further ours than as we choose them; *that all sin lies in the will, and all will implies choice;* that those thoughts, therefore, which are not our choice, which we reject with a settled aversion and abhorrence, will never be placed to our account. So that our thoughts, however indecent or irregular soever they may be, are rather to be considered the infirmities of our corrupt nature, than our sins, properly so called. If we close with any thought that prompts us to evil, so as to be pleased with it, to think of pursuing it till it be brought into action, in that case we can no longer plead our natural corruption; for in that very instant we become actual sinners, or actual transgressors of the law of God. The mind is passive in receiving its notices of things, whether pure or impure; but it is active in its determination whether to harbour or discard them. As far as it is passive, it is certainly innocent; as far as it is active, it is accountable: and it is constantly active when we dwell upon impure thoughts with complacency—when we strengthen ourselves in wickedness by cherishing the remembrance of guilty joys, and laying scenes in our imagination for the entertainment of future pleasures. Here, then, we see in what the government of our thoughts consists: they are not criminal till they have the consent of the will; and the soul can withhold that consent till it has sufficiently considered the whole case." . . . . .

"Notwithstanding what I have hitherto said concerning the diligence with which we are to keep our hearts, yet this is always to be remembered, that with our diligence we must be careful to join discretion. My meaning is this: we must have a care not to extend our thoughts immoderately, and more than our tempers will bear, even to the best things. And the way to do that is, not to put them too much or too long upon the stretch at any one time; but to relax them when there is occasion, and to let them run out and entertain themselves upon anything that comes to hand, so long as it is innocent."*

Burton frequently adverts to the recurrence of unholy and impure thoughts as a mental symptom of bodily disease, and so formidable a source of anguish as sometimes to occasion suicide.† Archbishop Secker, who was himself originally a physician, when speaking of "sin against the Holy Ghost," says: "As for what some good people are often terrified about, the wicked imaginations that come into their minds, and expressions that come out of their mouths, at times, almost whether they will or not, in proportion as they are involuntary, they are not criminal in them, *be they ever so bad*. . . . . . When they apprehend they cannot be pardoned, they entirely mistake their own case, either through ignorance or false opinions, or excessive tenderness of mind; or indeed more commonly by reason of some bodily disease, though perhaps unperceived by themselves, which depresses their spirits and clouds their understanding, and *requires the aid of medicine*."‡

Emboldened by such theological authorities—writers whose orthodoxy is above all suspicion—I would suggest that the attention of the psychologist should be particularly directed to the physical state of the organic functions of life, when he witnesses instances of an exalted or depressed condition of the religious feelings, different in their character from ordinary and healthy manifestations, and not clearly and distinctly traceable to legitimate influences. I am aware that there is a disposition on the part of those who take an *ultra spiritual* view of the mind's operations, to repudiate as blasphemy the material theory just enunciated. "What cheer," says Emerson, "can the religious sentiment yield, when that is suspected to be secretly dependent upon the seasons of the year and the state of the blood?" "I knew," he continues, "a witty physician, who found theology in the biliary duct, and used to affirm that if there was disease of the liver the man became a Calvinist, and if that organ was sound he became a Unitarian." In reply to this piece of pleasantry, I would observe that many a man has considered himself spiritually lost whilst under the mental de-

* "New Whole Duty of Man."

† "Anatomy of Melancholy."

‡ "Lectures on the Church Catechism."

pression resulting from long-continued hepatic and gastric derangement; and instances frequently occur of persons imagining themselves to be condemned to everlasting punishment, to be the subjects of demoniacal influence, and to hold personal converse with our Saviour, owing to the existence of visceral disease, or a congested condition of some one of the great vascular or nervous centres. In the former case the mind has been restored to a right and saving appreciation of Divine Mercy, and has been made to rejoice in comfort and hope, as the effect of a course of alterative medicine; and the morbid and unnatural ideas of demoniacal possession, and satanic and Divine presence, have vanished as soon as the bowels and various secretions have been made to act with healthy regularity, and the cupping-glasses have aided us in relieving the oppressed cerebral vessels. "It is probable," says Dr. Cheyne, "that they who have formed a lively conception of the personal appearance of Satan, from prints or paintings, have often had the conception realized in nervous or febrile diseases, or after taking narcotic medicine; and it is but charitable to believe that Popish legends, which describe victories over Satan, by holy enthusiasts, have had their origin in delusions of the senses, rather than that they were pious frauds."—"If it were," says Baxter, "as some fancy, a possession of the devil, it is possible that physic might cast him out. For if you cure melancholy, (black bile,) his bed is taken away, and the advantage gone by which he worketh. Cure the bile, and the choleric operations of the devil will cease: it is by means and humours in us that the devil worketh."

I am acquainted with an excellent Christian lady, who, at the critical period, loses all sense of religious impressions; her language during these attacks of partial derangement is most distressing and painful. I have occasionally to prescribe for a gentleman subject to attacks of sub-acute bronchitis, accompanied with a temporary perversion of the moral sense, owing, it is surmised, to the altered condition in the quality of the blood circulating in the brain. During these paroxysms his mind repudiates all idea of the existence of a God, and of a future state; and yet, when a healthy supply of properly arterialized blood is transmitted to the brain, the patient manifests, both in his conduct and conversation, the character of a true Christian gentleman.

The rationale of epidemic fanaticism is a subject of deep and important philosophic interest. How often mere exalted physical sensibility has been mistaken for the operation of the Holy Spirit; and illusions of the senses been faithfully and graphically recorded as evidences of Divine or satanic presence. Was not Luther, whilst in confinement, under the influence of temporary insanity? His representations—believed by many to this day in their literal sense—that he had frequent personal contests with the devil, most probably depended upon local cerebral congestion, or morbid state of the retina, and would, in our times, have justified a suspicion of the soundness of his mind. In the early history of the crusaders, and during epochs of religious and political commotion, such as the Reformation, and other social convulsions, it would not be difficult to cite numerous well-marked and unequivocal cases of insanity, which were considered at the time as instances of heroic devotion, political patriotism, and religious enthusiasm. Mr. Dendy has written so ably and lucidly on the subject of apparitions,* that I do not deem it necessary to more than refer to the connexion which we, as psychologists, know so closely exists between what are considered to be supernatural phenomena, and certain derangements of the cerebral circulation, diseases of the heart, and disorders of the alimentary canal and digestive organs. Dr. Ferriar observes: "Instead of regarding these ghost-stories with the horror of the vulgar, or the disdain of the sceptic, *we should examine them accurately, and should ascertain their exact relation to the state of the brain, and of the external senses.* The terrors of nocturnal illusions would then be dissipated, to the infinite relief of many wretched creatures; and the appearance of ghosts would be regarded in its true light, as a symptom of bodily distemper, and of little more consequence than a headache, and rigor attending a common catarrh."†

I have known cases in which a belief in the appearance of an apparition has ushered in, at an early age, severe brain-disease, and in advanced life has been precursory of paralysis, apoplexy, and insanity. A gentleman, as the effect of an active condition of the cerebral circulation, saw for several nights a ghastly spectre in his bedroom. A week afterwards he had an attack of apoplexy, of which he died.

It is our duty, as psychologists, to trace the relationship between certain palpable deviations from a normal state of thought, feeling.

* "Philosophy of Mystery." By W. C. Dendy, Esq.

† "Theory of Apparitions."

and action, often associated apparently with great vigour of understanding, brilliancy of genius, and power of continuous attention to the complicated and active business of life, and those states of the bodily health and physical organization which may originate and stimulate to action such morbid mental phenomena. There is much latent, undetected, and unrecognised insanity in real life: bringing with it a long train of deep and incurable misery. It assumes many aspects: occasionally it exhibits itself in the form of intemperance—an uncontrollable propensity for stimulants clearly having a mental origin—in extreme eccentricity, and in acts of a morbidly impulsive character. Again, it is manifested in brutal and cruel conduct; in others, it is evidenced either in an unnatural and unreasonable hatred of relatives, a total want of all moral sense, extreme irritability, tendency to crime, acts of viciousness, or in habits of inveterate lying. In fact, its shape is protean; and although those so unhappily afflicted often pass through life as sane, healthy, and rational persons, in the estimation of the Medico-psychologist they are suffering from disordered understandings, and ought to be brought within the sphere of remedial medicine.

The biography of tyrants, both REGAL and DOMESTIC, is yet to be written; and it remains for the philosophic historian, capable of appreciating the effects of defective and arrested cerebral organization,—the influence of physical and moral agents; and of bodily disease upon the character and temperament,—to account psychologically for the actions of men, the records of whose lives form the dark scenes of history, and present to the world a continuous career of morbid selfishness, crime, cupidity, caprice, tyranny, brutality, and vice. We do not possess data to enable us to judge satisfactorily of the mental or physical state of a Nero, a Caligula, or a Tiberius, who, as Tacitus informs us, was designated by his tutor, at the age of twenty, as "*a compound of mud and blood;*" but is it not charitable to suppose they were physically and morally diseased, and of unsound mind, the insanity manifesting itself in conduct, and not in ideas? Again, can we advance anything, as psychologists, in palliation of the crimes of Catherine de Medici?—or that would extenuate in the eyes of the world the brutal treatment to which Frederick William of Prussia, father of Frederick the Great, subjected his son?—or would be an apology for the atrocious tyranny and savage brutality of Judge Jeffreys?—anything to excuse the cold, calculating murders of Henry VIII.?—or the refined crimes of, and thirst for blood exhibited by Robespierre? —or say a word in extenuation of the unnatural furor with which the poet Savage was hunted to death by his own mother? Poor Savage! No sooner was he born than his mother discarded him. After he had discovered the name of his parent, it was his practice to walk in the dark evenings for several hours before the door of his mother's house, in the hope of seeing her as she might come by accident to the window, or cross her apartment with a candle in her hand; but Dr. Johnson says, "he could neither soften her heart nor open her hand." In attempting to explain the extraordinary hatred exhibited by the mother of Savage towards her only child, and the intense malignity with which she, by the most awful falsehoods, attempted to procure the execution of the unhappy poet, Dr. Johnson observes, that the "most execrable crimes are sometimes committed without apparent temptation." When alluding to his own miserable fate, Savage feelingly exclaims,—

> "No mother's care
> Shielded my infant innocence with prayer;
> No father's guardian hand my youth maintained,
> Called forth my virtues, or from vice restrained."

May not all these monstrous departures from ordinary and healthy modes of thought, impulse, and action, constitute evidence, not only of depravity and vice in their ordinary signification, but *of undetected, unperceived, unrecognised mental disease, in all probability arising from cerebral irritation or physical ill-health?* Catherine de Medici's disposition did not show itself until after the death of her husband. I am not in a position to say how much of her conduct was to be attributed to the shock of his dissolution: but it is said she suffered from determination of blood to the head, so severe in its character as to require occasional bleeding for its relief. Frederick William of Prussia was a debauchee and a drunkard. He conceived, without any reason, an inveterate hostility to his eldest sister, and to the prince, his son, afterwards Frederick the Great. He compelled them to eat the most unwholesome, disgusting, and nauseous articles of diet. He was in the habit of spitting in their food, and behaving towards his son with great ferocity. King Frederick suffered from severe attacks of hypochondriasis, and great mental depression, and it was during one of these paroxysms that he attempted suicide. Who can entertain a doubt of his insanity, or of the good that would have resulted had his brutality and cruelty been considered symptoms of some affection of the brain, and he had been treated accordingly? Robespierre, after his death, was found to have extensive visceral disease; and it is notorious he suffered much from this affection during life. It is recorded that he rolled on the ground for hours in acute pain.

Judge Jeffreys, it is said, was "tortured by a cruel internal malady, which had been aggravated by intemperance."* In the celebrated Western, or "Bloody" Assizes, this monster is said to have *hanged* 320 and *transported* 855 persons for "the most part of blameless life and of high religious profession!" Previously to his starting for the circuit Jeffreys' health and spirits had given way. "He had been deeply mortified by the coldness of the king and by the insolence of the chief justice, and could find little consolation in looking back on a life, not, indeed, blackened by an atrocious crime, but sullied by cowardice, selfishness, and servility." During the celebrated trial of Lady Alice Lisle, Jeffreys is said to "have stormed, cursed, and sworn in language which no well-bred man would have used at a race or cock-fight." Addressing himself to one of the witnesses who gave evidence in favour of Lady Alice, he exclaimed, with an oath, "Was there ever such a villain on the face of the earth? Dost thou believe that there is a God? Dost thou believe in hell-fire? A Turk is a saint to such a fellow as this! Oh, blessed Jesus! What a generation of vipers we live among! Was there ever such an impudent rascal? Hold the candle to him, that I may see his brazen face!" As Jeffreys proceeded in his bloody business, his "*spirits rose higher and higher as the work went on. He laughed, shouted, joked, and swore in such a way, that many thought him drunk from morning to night.*"†

I again ask, if the psychological physician is not best fitted, by thought, reflection, and education, to investigate, and elucidate satisfactorily, these interesting morbid psychical phenomena, and to suggest the possibility of effecting important changes in the *moral* and intellectual condition, by bringing within the sphere of medical treatment the *physical state* giving rise to these obvious departures from sound and normal manifestations of the affections and propensities? *Many a suicide would be prevented, and murderous and criminal impulse destroyed, if an active*

* Macaulay's "History of England," vol. i. p. 67.
† Macaulay, p. 600.

*cathartic could be exhibited, or the cerebral circulation relieved, and rendered less active by means of local depletion.* There are crimes for which men have been hanged, which might have been prevented by physical treatment. Damien persisted in declaring to the last that had he been bled in the morning, as he wished and requested, he never would have attempted the assassination of Louis XV. It is recorded of Caligula, that his reign commenced with mildness, but that the end of the first year, *after a violent attack of bodily illness,* he commenced his career of cruelty, violence, and crime, slaughtering the noblest men of Rome, and hunting the spectators of a public show into the waters of the Tiber!

Is it not possible, by a course of medicine and a system of dietetics, to modify the diathesis, both mental and physical? Dr. Arbuthnot says he cured an irascible diathesis by enforcing a milk and vegetable diet, and Dr. Rush relates a case of a man who was subject to severe paroxysms of anger, who was cured by the application of leeches to the head.* Let it not be thought for a moment that I suppose the skill of the *physician* can supersede the aid of the *divine;* but "*the service of God is a* REASONABLE *service;*" and divines themselves, eminent for piety and learning, are not unfrequently subjected to medical treatment, not only to arrest aberrations of the intellect, but to cure perversion of the moral sentiments.

In referring to the possibility of the hallucinations of Luther being the result of physical causes, Coleridge observes that his unremitting activity, labour, and sedentary mode of life, during his confinement in the Wartzburg, had undermined his former usually strong health. Luther suffered from many of the most distressing effects of indigestion, so much so that his friend, Melancthon, urged him to consult the physicians of Erfurth. He did so, and for a time regained his health; he soon, however, relaxed into his former habit. Coleridge says it was evident

* An English traveller calling on Voltaire, at Ferney, found him desponding, grumbling, and dissatisfied with all mankind. The conversation soon fell upon the miseries of life, and the Frenchman's *ennui* and the Englishman's spleen exalting the mutual discontent of both parties, they ended by deciding that existence was too grievous a burden to be borne any longer, and agreed to commit suicide together on the following morning. The Englishman, punctual to his engagement, arrived at the appointed hour, provided with the means of destruction; but the volatile Frenchman was no longer in the same miserable, suicidal mood, for on the other proposing to proceed immediately to the execution of their project, Voltaire laughingly replied, "Pardonnez-moi, Monsieur, mais mon lavement a très bien opéré ce matin, et cela a changé toutes idées-là."

from his letters that Luther suffered from great irritability of the nervous system, the common effect of deranged digestion in men of sedentary habits, who are, at the same time, intense thinkers; and this irritability, added to a revivification of the impressions made upon him in early life, and fostered by the theological system of his manhood, is abundantly sufficient to explain all his apparitions and all his nightly combats with evil spirits. "I see nothing," says Coleridge, "improbable that in one of those unconscious half sleeps, or rather those rapid alternations of the sleeping with the half-waking state, which is the '*true witching time,*'

'The season
Wherein the spirits hold their wont to talk.'

the fruitful *matrix* of ghosts,—that in one of those moments of slumber, into which the suspension of all thought, in the perplexity of deep thinking, so often passes, Luther should have had a full view of the room in which he was sitting, of his writing-table, and, at the same time, a brain image of the devil, vivid enough to have acquired an apparent *outness*, and a distance regulated by the proportion of its distinctness to that of the objects really impressed upon the outward senses."*

No one can read the interesting account of the unhappy controversy between Hume and Rousseau, prefixed to the first volume of the historian's *Philosophical Essays,* without having the conviction forced upon the mind, that Rousseau must have suffered, at the time, from temporary insanity. "The strange influence of his bodily temperament on his understanding; his constitutional melancholy, pampered into a morbid excess by solitude; his wild dreams of suspicion; his hypochondriacal fancies of hosts of conspirators, all leagued against him and his cause, and headed by some arch-enemy, to whose machinations he attributed every trifling mishap," are referred to as indications of an abnormal state of mind, not at the period recognised, or urged as some excuse for conduct which set the author of *Emelius* against all the world, and all the world against him. The persecution which Rousseau appeared to court, his affectation of singularity, his determination to live in a world of his own creation, and to have no sympathy or thought in common with his fellow-men,—all indicate a constitution of mind, if not actually diseased, at least not remotely removed from that condi-

* "The Friend," p. 238.

tion. Such, it would appear, is destined to be the unhappy fate of all who, to gratify a morbid singularity, resolutely oppose their own crude notions to the calm, deliberate, and healthy judgment of the rest of the world. In attempting a philosophical explanation of these psychical phenomena, Coleridge observes: "To know that we are in sympathy with others, moderates our feelings, as well as strengthens our convictions; and for the mind which opposes itself to the faith of the multitude, it is more especially desirable that there should exist an object *out of itself*, on which it may fix its attention, and thus balance its own energies."*

There are other important subjects that come legitimately and almost exclusively within the range of the speculations of the psychological physician, to which I can only cursorily refer. It is his duty to investigate the moral as well as physical effects of climate, and of the different systems of dietetics, upon the psychical character of nations; the laws relating to the influence of the mind of both parents on the offspring; the transmission of hereditary diseases and mental qualities; the nature of the education best adapted to strengthen the mind and avert the development of insanity; the influence of different kinds of amusements upon the public morals; the effect of the prevailing literature upon the formation of character and the development of the human mind; the effect of different kinds of pursuits upon the mind and character; and the *modus operandi* of music as a remedial agent. The interesting and important points involved in the investigation of the subjects of crime, penal legislation, capital punishment, trance, somnambulism, dreaming, &c., are only to be solved by the philosopher who, to an enlarged and cultivated understanding, unites a knowledge of the higher departments of physiology, joined to an acquaintance with the science of mental philosophy. Need I advance another argument to demonstrate the imperative necessity of establishing, in connexion with our national universities, a professorship of medical psychology, for the special investigation of these essentially necessary sections of philosophical inquiry, so important to the PHYSICIAN, the DIVINE, the LEGISLATOR, the JURIST, the EDUCATOR OF YOUTH, and to all who feel, as all the educated classes should feel, an interest in the intellectual and moral progress, the temporal and eternal welfare, of man?

* "The Friend," p. 224.

Finally, I would observe, that of all the subjects that can occupy the attention of the philosophic physician, none equals in importance or in grandeur those which I have had the honour of recommending to your special attention. What can compare in dignity, in sublimity, in comprehensiveness, or in the lofty aim of its disquisitions, to the study of the nature and operation of that spiritual essence, upon the right knowledge and cultivation of which depends our happiness, both in time and in eternity? As the mind advances in a knowledge of its own phenomena, the intellect expands, new sources of delight open to us, and the pleasure we experience in the pursuit of these exalted speculations impresses forcibly upon the mind itself conclusive evidence of its own DIVINITY. He who has habituated himself to trace out the numerous applications of mental philosophy to the important subjects of education, morals, and legislation; to analyse the nature of thought, the laws regulating the association of our ideas, the springs of action, the origin of our happiness, the laws of moral science, the nature of the passions, the formation of character, the foundation of our hopes, and the influence of our emotions,—will appreciate the value of this branch of science. The physician will be conscious, as he advances in a knowledge of the constitution of the mind, that his love of truth is growing strong; and whilst, in the spirit of true humility, he acknowledges the limited nature of his intellectual powers, he will, whilst contemplating their grandeur and importance, recognise the GOODNESS AND MAJESTY OF GOD.

In glancing retrospectively at the preceding portion of this lecture, I feel oppressed by a consciousness of the imperfect and inadequate manner in which I have sketched the exalted spiritual functions of the physician. Have I not reason to blame myself for attempting to grasp a subject so great and sublime? And have not those whom I have the honour of addressing a right to censure me for my presumption in selecting for illustration a theme requiring for its successful elucidation and expansion an amount of knowledge of the higher departments of philosophy, an originality of conception, and power of illustration, to which I have no pretension? Our position as medical philosophers, occupied in the investigation of the phenomena of life, of mind, and of disease, entails upon us anxious, solemn, and responsible duties. In the hour of pain, when the spirit is humbled by suffering—in the day of distress—in the solemn moment of dissolution,—it is our high and noble privilege, like guardian angels, to hover about the couch of the sick and the dying. We enter the chamber of the man writhing with agony, bereft, perhaps, of that which alone made existence pleasurable, the right exercise of the mental powers, and loud and affectionate demands are made upon our sagacity and skill. Life—the silken thread, the silver cord of life—depends upon our rapid appreciation of the phenomena of disease, and ready administration of remedial agents for their relief and cure. Our profession is a noble one—a most dignified, exalted, and honourable calling. "The skill of the physician puts in requisition the highest faculties of the human intellect, as its administration calls forth the tenderest sympathies of the human heart. The able and kind physician is a human benefactor. He garners up the treasures of learning and experience, that he may dispense them again to his suffering brethren. He comes with his timely succour, cheering both body and spirit with the single boon of health. He raises the sick man from his couch of pain, and sends him forth, elate and vigorous, for fresh enjoyment. He restores the ailing, and rejoices their despondent friends. He gives new life to the sick, and revives the hopes of those who depend on the sick man's recovery for subsistence."*

While feeling that the best of our works are imperfect, and that we must rely for our future happiness upon the great mercy of God, and not upon our own merits, I cannot be forgetful how great is our responsibility for the right use of our talents, and the faithful discharge of our solemn and anxious duties; and I would therefore venture, with all deference and respect, to address to each of my professional brethren the admonition of one of America's most gifted poets:†

> "So live, that when thy summons comes to join
> The innumerable caravan, that moves
> To that mysterious realm, where each shall take
> His chamber in the silent halls of Death,
> Thou go not, like a quarry-slave at night,
> Scourged to his dungeon; but, sustained and soothed
> By an unfaltering trust, approach thy grave
> LIKE ONE WHO WRAPS THE DRAPERY OF HIS COUCH
> ABOUT HIM, AND LIES DOWN TO PLEASANT DREAMS."

---

* "The Girlhood of Shakspeare's Heroines," chapter—The Vocation of the Physician. By Mrs. Mary Cowden Clarke.

† William C. Bryant.

